# The Impact of the Antibiotic Fosfomycin on Wastewater Communities Measured by Flow Cytometry

**DOI:** 10.3389/fmicb.2021.737831

**Published:** 2022-03-03

**Authors:** Shuang Li, Zishu Liu, Christine Süring, Luyao Chen, Susann Müller, Ping Zeng

**Affiliations:** ^1^Department of Environmental Microbiology, Helmholtz Centre for Environmental Research – UFZ, Leipzig, Germany; ^2^College of Environmental and Resource Sciences, Zhejiang University, Hangzhou, China; ^3^Institute of Water Ecology and Environment, Chinese Research Academy of Environmental Sciences, Beijing, China

**Keywords:** fosfomycin, spread of antibiotics, microbial community dynamics, ecology of microbial communities, single cell analytics, bio-augmentation

## Abstract

Fosfomycin is a re-emergent antibiotic known to be effective against severe bacterial infections even when other antibiotics fail. To avoid overuse and thus the risk of new antibiotic resistance, the European Commission has recommended the intravenous use of fosfomycin only when other antibiotic treatments fail. A release of fosfomycin into the environment *via* wastewater from not only municipalities but also already from the producing pharmaceutical industry can seriously undermine a sustaining therapeutic value. We showed in long-term continuous-mode bioreactor cultivation and by using microbial community flow cytometry, microbial community ecology tools, and cell sorting that the micro-pollutant altered the bacterial wastewater community (WWC) composition within only a few generations. Under these conditions, fosfomycin was not readily degraded both at lower and higher concentrations. At the same time, operational reactor parameters and typical diversity parameters such as α- and intracommunity β-diversity did not point to system changes. Nevertheless, an intrinsic compositional change occurred, caused by a turnover process in which higher concentrations of fosfomycin selected for organisms known to frequently harbor antibiotic resistance genes. A *gfp*-labeled *Pseudomonas putida* strain, used as the model organism and a possible future chassis for fosfomycin degradation pathways, was augmented and outcompeted in all tested situations. The results suggest that WWCs, as complex communities, may tolerate fosfomycin for a time, but selection for cell types that may develop resistance is very likely. The approach presented allows very rapid assessment and visualization of the impact of antibiotics on natural or managed microbial communities in general and on individual members of these communities in particular.

## Introduction

Worldwide, depending on the antibiotic class, 20–80% of antibiotics enter the environment *via* urine and feces as well as *via* the deliberate or accidental release (European Medicines Agency, 2020), e.g., by production facilities of the pharmaceutical industry (Andersson and Hughes, 2014). Antibiotics in wastewater treatment plants (WWTPs) have been shown to select for antibiotic-resistant bacteria (ARBs) and to facilitate horizontal gene transfer (HGT) of antibiotic resistance genes (ARGs) to previously antibiotic-sensitive bacteria (Brown and Wright, 2016). The host range of ARGs in highly diverse wastewater environments is huge (Che et al., 2019). Although the number of potential antibiotic-resistant pathogens (ARPs) has been found to decrease from the inflow to the effluent in a WWTP, acquired resistance is spreading between wastewater environments and ecosystems (Andersson and Hughes, 2014; Hernando-Amado et al., 2019; Hou et al., 2019). ARBs and the spread of ARGs have been described to affect the structure and function of microbial consortia in managed ecosystems such as WWTPs or natural ecosystems such as lakes, surface water, and soil, at least in the vicinity of effluent discharge (Chu et al., 2018).

Fosfomycin (cis-1,2-epoxypropylphosphoric acid) is a naturally occurring antibiotic and was first isolated from the fermentation broth of *Streptomyces fradiae, Streptomyces virichromogens*, and *Streptomyces wedmorensis* in 1969 (Hendlin et al., 1969; Silver, 2017; Aghamali et al., 2019). Glamkowski et al. (1970) chemically synthesized fosfomycin in 1970. The antibiotic is a small (138 Dalton), strongly polar, thus highly soluble molecule of a so far unique class with rapid diffusion abilities (Bergogne-Bérézin, 2005; Raz, 2012). Fosfomycin acts during the early stage of cell wall synthesis both in Gram-positive and Gram-negative bacteria (Aghamali et al., 2019). It is currently considered a valuable re-emergent antibiotic with broad-spectrum antibacterial activity and effective also against multidrug-resistant isolates (Cao et al., 2019). However, in industrial production, similar to the production of other antibiotics, fosfomycin is disposed of in wastewater and inefficiently removed by conventional wastewater treatment systems before being released into the environment. Concentrations far above average (18 mg L^–1^ fosfomycin) can temporarily be measured in the inflow of a pharmaceutical WWTP, and concentrations up to 10 mg L^–1^ could be expected in the vicinity of WWTP effluents (Zeng et al., 2014). Already a metallo-β-lactamase producing *Escherichia coli* strain has been found in the effluent of a WWTP connected to a canal in Tokyo Bay, Japan, which carried multiple antimicrobial resistance genes including fosfomycin, posing a potential threat to human health and ecosystem safety (Sekizuka et al., 2019). Numerous studies have been conducted on the development of proper treatment technologies for the removal of fosfomycin, both physical and chemical processes, but their relatively high costs hinder bigger-scale application (Xie et al., 2019). Thus, most of the larger fosfomycin manufacturers rely on biological processes to treat pharmaceutical wastewater.

High resolving single-cell Raman spectroscopy or metagenomics allows for tracking the fate of ARGs in WWTPs (Li et al., 2020; Brito, 2021). Especially the long-read nanopore sequencing technology has been described to successfully detect ARP profiles in less than 24 h after receiving a sample (Che et al., 2019). However, these highly resourceful approaches, which detect the transfer and spread of ARGs in bacterial communities, are still widely untapped to study the effects of antibiotics on antibiotic-susceptible bacteria in wastewater. A similar fast and underused technology based on single cell analytics is microbial community flow cytometry, which provides powerful means for monitoring structural and functional changes of complex communities with high temporal resolution (Koch et al., 2015; Liu et al., 2019; Vucic et al., 2021). As a low-cost observation method, flow cytometry measures the light scatter and fluorescence characteristics of every single cell in a community sample and allows the precise quantification of cells with similar phenotypical properties (Koch et al., 2013; Cichocki et al., 2020).

The aim of this study was to investigate if and to what extent fosfomycin can be biologically degraded or tolerated by a typical wastewater microbial community (WWC) and if the antibiotic has an impact on the structure and function of a WWC. Flow cytometry was used as a tool to follow dynamic changes of arising and disappearing subcommunities to reveal diverging and segregated responses of cell subsets of a WWC to fosfomycin. In parallel, a bio-augmented (*gfp*-labeled) *Pseudomonas putida* strain as a typical member of WWTPs (Chu et al., 2018) with inherent resistance to fosfomycin (Falagas et al., 2019), and as a possible future chassis for fosfomycin biodegradation pathways (Pallitsch et al., 2017; Nikel and de Lorenzo, 2018), was used as a model and marker organism, and its fate was continually tracked in a WWC under fosfomycin pressure. Exemplary WWCs and dominant cytometrically sorted subcommunities were analyzed taxonomically based on the 16S rRNA gene to identify phenotypes that may have been particularly selected by fosfomycin. The study provides evidence of the impact of fosfomycin on microbial communities in WWTPs and points out potential pitfalls if bio-augmented bacteria were to be used for antibiotic biodegradation.

## Materials and Methods

### Cell Cultivation

#### Cultivation of the *gfp*-Labeled *Pseudomonas putida* KT2440

The *gfp*-labeled *P. putida* strain has a chromosomally located *gfp*-gene (P_*A*1/04/03_-RBSII-*gfp*mut3*-T0-T1 transposon cassette) at random position (Heydorn et al., 2000; Dechesne et al., 2005), which presented an insignificant metabolic burden (Lambertsen et al., 2004). The strain was precultivated on LB agar plates (Lysogeny broth, 30°C, 72 h). For liquid culture, a colony of the strain was inoculated into a 100-ml LB medium (500 ml flasks, 30°C, 125 rpm, 192 h). The LB medium contained tryptone 10 g L^–1^ (Oxoid, Hampshire, United Kingdom), yeast extract 5 g L^–1^ (Difco, Detroit, MI, United States), and NaCl 5 g L^–1^. Growth parameters and GFP autofluorescence of the strain were analyzed *via* OD_600, λ = 5 mm_ (Ultrospec 1100pro, Amersham Biosciences, Buckinghamshire, United Kingdom) (Supplementary Figure 1.1) and flow cytometry (excitation of GFP fluorescence at 488 nm, emission at 530/40 nm, additional staining of cells and single cell analysis techniques below). Samples were taken for up to 192 h in biological triplicates. The strain showed an uncoupled DNA synthesis (Müller, 2007) within the first hours of cultivation and reached the stationary phase after 24 h by still representing four subpopulations with different chromosome numbers (C1n-C8n). The typical bi-modal DNA distribution (C1n-C2n) was reached after about 120 h (Supplementary Figure 1.2). The gfp-autofluorescence was the highest during exponential growth and declined gradually during the 192 h of cultivation due to stationary phase conditions, but was always clearly detectable within this period of time (Supplementary Figure 1.3).

#### Origin and Cultivation of the Wastewater Microbial Community

The WWC was obtained from an activated sludge basin of a full-scale WWTP in Eilenburg (Germany, 51°28′29.7″N; 12°37′13.9″E), fractionated and stored at −20°C. To start an experiment, WWC fractions were slowly defrosted and cultivated in a LB liquid medium. For the batch experiments, equal amounts of these fractions were added to each flask at an initial optical density (OD) of about 2 (OD_600, λ = 5 mm_, Ultrospec 1100pro, Amersham Biosciences, Buckinghamshire, United Kingdom) and cultivated in a 100-ml medium (500-ml flask, 30°C, 125 rpm, 72 h). The continuous reactor experiment was started with an OD of about 0.085 after inoculation of 10 ml of a 72-h batch culture of a WWC (detailed below).

#### Bio-Augmentation With *gfp*-Labeled *Pseudomonas putida* KT2440

The survival potential of *gfp-*labeled *P. putida* KT2440 in batch-grown WWCs was followed by (1) tracking the *gfp*-labeled *P. putida* within WWCs using flow cytometry, (2) changing the cell proportions of bio-augmented *gfp*-labeled *P. putida* within WWCs, and, on the contrary, (3) tracking the change in WWCs due to different bio-augmentation proportions of *P. putida*. For bio-augmentation of 72-h batch-grown WWCs, exponential-phase *gfp*-labeled *P. putida* KT2440 cells were used. Specifically, 6-h grown cells of *gfp*-labeled *P. putida* KT2440 were mixed in proportions of 1, 10, and 50% with the batch-grown WWC, based on OD (OD_600, λ = 5 mm_, Ultrospec 1100pro, Amersham Biosciences, Buckinghamshire, United Kingdom), respectively. The cells of the WWC were harvested and centrifuged (sterile; 3,200 g, 10 min, 4 °C). For 1% proportion of *gfp*-labeled *P. putida* KT2440, the WWC co-culture was resuspended in a fresh 100-ml LB medium, while for 10 and 50% proportions, the WWC was directly added to batch-grown *gfp*-labeled *P. putida* KT2440. All WWC co-cultures were grown at 30°C, 125 rpm for 192 h in triplicates, respectively (Supplementary Information 2).

For the bio-augmentation of the continuously grown bioreactor WWC, 24-h batch-grown *gfp*-labeled *P. putida* KT2440 cells were used. For harvesting, the *gfp*-labeled *P. putida* KT2440 cells were centrifuged (sterile; 3,200 *g*, 10 min, 4°C), and two-thirds of the supernatant were removed. The cells were vortexed (IKA^®^ Vortex Genius 3, IKA^®^-Werke GmbH & CO.KG, Germany) within the remaining solution. The accurate proportion for augmentation of the continuously operated bioreactor was calculated based on OD (OD_600, λ = 5 mm_, Ultrospec 1100pro, Amersham Biosciences, Buckinghamshire, United Kingdom) and culture volume (V) added according to Equation 1:


(1) $$\begin{array}{c} \mathrm{proportion}\mathrm{of}\mathrm{augmentation}\left(\%\right)\\ =\frac{\text{OD}\left(P.putida\right)\times V\left(P.putida\right)}{\text{OD}\left(P.putida\right)\times \text{V}\left(P.putida\right)+\mathrm{OD}\left(\text{WWC}\right)\times V\left(\mathrm{WWC}\right)} \end{array}$$

#### Continuous Cultivation of the Microbial Community

The continuous cultivation was performed to provide a balanced growth situation for studying the dynamics of a complex WWC under fosfomycin pressure during a long-time experiment for 672 h. The continuous cultivation was carried out in a 1-L Biostat^®^ MD laboratory stirring bioreactor (BIOSTAT B-DCU II, Sartorius, Göttingen, Germany) with a final working volume of 800 ml. Operational conditions were set as in a previous study (Liu et al., 2018). In brief, the dilution rate was adjusted to *d* = 0.045 h^–1^ (0.6 ml min^–1^ LB medium: 36 ml h^–1^), which led to a generation time (τ) of 15.4 h and a hydraulic retention time (HRT) of 22.2 h. The dilution rate was maintained by a peristaltic pump (Watson Marlow, 101U/R, Falmouth, United Kingdom) during the whole experiment. The pH was kept constant at pH = 7.0 by adding 1M KOH or 1M H_2_SO_4_ as required and measured online in the liquid phase (Ingold, Mettler Toledo GmbH, Giessen, Germany). Temperature, stirring rate, and aeration were kept constant at 22°C, 400 rpm, and 2.0 L compressed air (1.2 bar) min^–1^ (Supplementary Figure 3.1). In addition to the *online* monitoring of the above parameters, samples were taken routinely at 28 time points for the measurement of cell number (CN), OD, chemical oxygen demand (COD), and fosfomycin concentration (FOM).

After inoculation with 10 ml (OD = 5.705) WWC, the experiment was started and subgrouped into four phases (Table 1). After an initial adaptation (phase 1: 0–168 h), proportions of 0.5% at 168 h and 2.5% at 264 h of *gfp*-labeled *P. putida* KT2440 were augmented into the continuous bioreactor (phase 2: 168–337 h). In phase 3 (337–432 h), a proportion of 2.5% *gfp*-labeled *P. putida* KT2440 was added at 337 h, and the LB medium was fed into the bioreactor together with the premixed antibiotic fosfomycin (10 mg L^–1^). In phase 4 (432–672 h), proportions of 2.5% *gfp*-labeled *P. putida* KT2440 were added at 432 and 529 h, and the concentration of fosfomycin was increased to 268 mg L^–1^ at 432 h. The 268 mg L^–1^ FOM was related to ½ of the half maximal effective concentration (EC 50) for fosfomycin (Xie et al., 2014). Daily, 13-ml samples were taken using a sterile syringe for the analysis of FOM, CN, OD, and COD as well as for flow cytometry of live *gfp*-labeled *P. putida* and for treatment of cells for flow cytometric measurement of community dynamics (Table 1 and Supplementary Information 3, 4).

**TABLE 1:** Continuous cultivation: times of augmentation of *gfp*-labeled *P. putida* KT2440 (in % proportions to the WWC) and of fosfomycin (FOM, mg L^–1^).

Phase	Time (h)	Concentration of FOM (mg L^–1^)	*gfp*-labeled *P. putida* addition	
			Time (h)	Proportion (%)
First	0–168	0	–	–
Second	168–337	0	168	0.5
			264	2.5
Third	337–432	10	337	2.5
Fourth	432–672	268	432	2.5
			529	2.5

*The color marks the subgroups for phases according to the addition of gfp-labeled P. putida KT2440 and FOM.*

### Analyses of Bulk Parameters

#### Analysis of Optical Density and Chemical Oxygen Demand

OD_600, λ = 5 mm_ was measured at the Ultraspec 1100pro (Amersham Biosciences, Buckinghamshire, United Kingdom). COD was measured with NANOCOLOR^®^ test tubes (Macherey-Nagel GmbH, Düren, Germany) following the manufacturer’s instruction manual. The measurement of COD followed the APHA (Federation, Water Environmental and APH Association, 2005) protocol. All samples were measured in triplicate.

#### Analysis of Fosfomycin Sodium

The concentration of fosfomycin sodium (FOM) was measured in the liquid phase. Liquid samples were sampled and centrifuged at 5,000 *g* (5 min, 4°C, Heraeus Fresco 21, Thermo Scientific, Germany). The supernatants were filtered using a 0.2-μm filter (Labsolute, Lot. 7042868, Germany), and the filtered fluids were collected in Falcon tubes (12 ml, Nunc conical centrifuge tubes, Thermo Scientific, Germany) and stored under −20°C until analysis. For the analysis, DIONEX ICS-2100 (Thermo Scientific Dionex, United States) was used, and data analysis was performed using the Chameleon 6.8 chromatography workstation (Thermo Scientific Dionex, United States). The mobile phase was 30 mmol L^–1^ KOH, and the flow rate was maintained at 1 ml min^–1^. Chromatographic separations were performed at 30°C on a Dionex IonPac™ AS11-HC anion column (250 × 4 mm, Thermo Scientific Dionex, United States). ASRS500 was used as an anion suppressor (4 mm, Theromo Scientific Dionex, United States) with a current of 75 mA. The injection volume was 25 μl. The typical retention time for fosfomycin was 3.8 min.

#### Analysis of Cell Number

For CN analysis, 1-ml sample was taken and 500-μl ice cold 50% glycerol [1:1 (v:v), pure glycerol : Millipore water] was added to a final glycerol concentration of 16.7%. The sample was kept at −20°C until cell counting. Before cell counting, the samples were thawed on ice and diluted with 0.85% saline solution to an estimated range of 10^9^–10^10^ cells ml^–1^. SYTO^®^9 dye (Lot. 2088729, Thermo Fisher Scientific, Eugene, OR, United States) was prepared as a 35-μM primary solution. For staining, SYTO^®^9 primary solution was added to the diluted sample to a final concentration of 1.75 μM and incubated for 15 min at room temperature (RT) before cell counting.

The cell counts were measured using the flow cytometer CyFlow^®^Space (Sysmex Partec GmbH, Görlitz, Germany) with the True Volumetric Absolute Counting mode measuring cells in a fixed volume of 200 μl. The flow cytometer was equipped with a 488-nm argon laser (50 mW, SAPPHIRE 488-50, Coherent, Santa Clara, CA, United States) for the excitation of forward scatter (FSC) and side scatter (SSC, trigger) and green fluorescence. For the daily optical calibration of the cytometer in the linear range, 0.5-μm yellow-green fluorescent FluoSpheres (ThermoFisher Scientific, F8827, Waltham, MA, United States) and 1.0-μm yellow-green fluorescent FluoSpheres (ThermoFisher Scientific, F13081, Waltham, MA, United States) were used. The measuring rate was adjusted to below 1,500 events s^–1^ *via* diluting samples with cell-free Millipore water. Based on a defined cell gate in an FSC vs. FL2 2D-plot, CNs per milliliter were counted automatically using the software FloMax (V2.4, Sysmex Partec GmbH, Germany). All samples were measured in triplicate.

### Analysis of Cell Characteristics by Flow Cytometry

#### Treatment of *gfp*-Labeled *Pseudomonas putida* KT2440

The *gfp*-labeled *P. putida* KT2440 cells were both directly measured as vital cells and after sampling, fixation, and staining of the WWC using flow cytometry (details below). The strain was detectable in the green range throughout the batch-cultivation period (192 h, Supplementary Figure 1.3), with slightly reduced fluorescent intensity at the end due to the less active physiological state of cells at the end of the stationary growth phase.

#### Treatment of the Wastewater Community

After harvesting, the samples were centrifuged at 3,200 *g* (10 min, 4°C). The supernatant was removed, and the cell pellet was resuspended in 2 ml paraformaldehyde solution (PFA, 2% in PBS) and incubated for 30 min at RT. Afterward, the cells were centrifuged again (3,200 *g*, 10 min, 4°C), resuspended in 4 ml 70% ethanol, and stored at −20°C. For staining, an aliquot of the fixed sample was washed twice with PBS (3,200 *g*, 10 min, 4°C) and then adjusted with PBS to an OD_700 *nm*_ of 0.035. Two milliliters of the adjusted cell solution was centrifuged (3,200 *g*, 10 min, 4°C), resuspended in solution B [0.24 μM DAPI in phosphate buffer (289 mM Na_2_HPO_4_ and 128 mM NaH_2_PO_4_ in distilled water)], and incubated overnight at RT.

#### Cytometric Analysis of the Cells

The cells were measured using a MoFlo Legacy cell sorter using the software Summit V4.3 (Beckman-Coulter, Brea, CA, United States) and evaluated according to previously published pipelines (Liu et al., 2019; Cichocki et al., 2020). The instrument was equipped with a 488-nm argon laser (400 mW, Coherent, Santa Clara, CA, United States) and a UV laser (355 nm, 150 mW, Xcyte CY-355-150, Lumentum, Milpitas, CA, United States). The 488-nm laser light was used for the detection of the FSC (488/10 nm band pass), the SSC (488/10 nm band pass, trigger signal), and the *gfp*-labeled *P. putida* KT2440 (GFP, 530/40 nm band pass). The DAPI fluorescence (DAPI, 450/65 nm band pass) was measured using UV excitation. The fluidic system was run at constant sheath pressure of 56.0 psi with a 70-μm nozzle. The sample pressure was adjusted within the range of 55.8–56.2 psi and around 3,500 events s^–1^. The sheath fluid was composed of a 10-fold sheath buffer (19 mM KH_2_PO_4_, 38 mM KCl, 166 mM Na_2_HPO_4_, 1.39 M NaCl in Millipore water) and further diluted with Millipore water to a 0.2-fold 0.13-μm filtered working solution (for cell sorting: 0.5-fold). For the daily optical calibration of the MoFlo in the logarithmic range, 0.5- and 1.0-μm UV Fluoresbrite Microspheres (both from Polysciences, Cat. No. are 18339 and 17458, Warrington, PA, United States) were applied. Furthermore, to ensure the daily reliability of the cell fixation and staining procedures, a microbial cytometric mock community (mCMC, Cichocki et al., 2020) was applied. The cells of the mCMC were handled identically to the staining protocol mentioned above. Prior to measurement, DAPI-stained cell samples were filtered to remove larger particles by using a nylon filter (CellTrics^®^ 50 μm, Sysmex Partec GmbH, Görlitz, Germany) and were spiked with 0.5- and 1-μm UV Fluoresbrite Microspheres (both Polysciences, 18339 and 17458, Warrington, PA, United States). The microspheres served as internal standards to monitor instrument stability and to allow for sample comparison over longer time periods. Cell data were collected in logarithmically scaled DAPI vs. FSC 2D-plots. Two cell gates were defined (for batch- and continuously grown WWCs, each), which comprised 200,000 virtual cells for each measurement. Apparent cell clusters were gated, and all defined gates were combined together to create the gate templates for batch- and continuously grown WWCs (Supplementary Figures 4.1, 4.2). The relative cell abundance of each subcommunity was computed with the FlowJo software (V10, FlowJo LLC, OR, United States) for batch-grown WWCs (G1-G33) and Summit V4.3 (Beckman-Coulter, Brea, CA, United States) for continuously grown bioreactor WWCs (G1–G31). Cytometric data were deposited at the FlowRepository database (Repository ID: FR-FCM-Z46E) under^1^.

#### Cell Sorting

Cell sorting was performed according to a previous published protocol (Cichocki et al., 2020) using the most accurate single- and one-drop mode (99% purity). A total of 500,000 cells of each selected gate were sorted into a 1.5-ml Eppendorf tube at an event rate not more than 1,500 events s^–1^. Sorted cells were harvested from the sheath buffer by centrifugation (20,000 *g*, 6°C, 25 min), and the cell pellets were stored at −20°C for subsequent DNA isolation.

#### Bioinformatics Evaluation

A flow cytometric 2D-plot represents 200,000 cells per analysis, characterized by FSC, SSC, and fluorescent parameters. Cells with similar optical properties cluster together as a subcommunity (i.e., gate). The positions and the numbers of gates as well as the cell abundances per gate reflect the microbial community structures (i.e., the community fingerprint). These cell abundances were evaluated by using the flowCyBar^2^. Data analysis and visualization were performed in RStudio (V1.2.1335, Boston, MA, United States) with R (V3.6.3, R Core Team, 2020) using R packages “vegan” (V2.5.6) for NMDs analysis, “Hmisc” (V4.4.0) for correlation analysis, and “ggplot2” (V3.3.0) for plotting.

### Determination of Diversity Values

#### Cytometric α- and β-Diversity Values

The cytometric α-diversity was calculated by gate-based Hill numbers (Dq = 2, also termed as the inverse Simpson index, Hill, 1973) using a procedure from Liu et al. (2018). Only those subcommunities were counted as dominant subcommunities that pass the average cell abundance threshold of 1/number of gates (3.03% for batch-grown and 3.23% for continuously grown WWCs). Cytometric intracommunity β-diversity values give information on community evolution by counting the numbers of unique subcommunities between successive sampling days of the same community. In this study, intracommunity β-diversity indicated time-dependent community variations in batch experiments as well as influences of fosfomycin disturbances in the continuous bioreactor experiment. Cytometric intercommunity β-diversity values were calculated by counting the numbers of subcommunities that were unique between the same sampling days of different communities (Liu et al., 2019). These values highlighted the differences between communities in the batch experiments dependent on the degree of bio-augmentation.

#### Nestedness and Turnover

The partitioning of β-diversity (βSOR, Sørensen pairwise dissimilarity) to nestedness (βNES) and turnover (βSIM) components was conducted using the “betapart” R package. For batch experiment, βNES and βSIM were analyzed intracommunity pairwise at successive time points and intercommunity pairwise at different augmentation proportions using the function “beta.pair.” For the continuous bioreactor experiment, samples from phase 4 (432–672 h) were analyzed intracommunity wise using the function “beta.multi” (Baselga, 2010; Liu and Müller, 2020).

### 16s rRNA Gene Amplicon Sequencing

Whole community samples and flow cytometrically sorted subcommunity samples from the continuous bioreactor experiment were further investigated by 16S rRNA gene amplicon sequencing. Whole community samples were harvested at 1, 432, and 550 h, and cells of sorted subcommunities were obtained at 550 h. DNA was extracted using 70 μl of 10% Chelex 100 solution (Bio-Rad, Hercules, CA, United States) according to a protocol from Cichocki et al. (2020). 16S rRNA gene (V3–V4 region) amplicon sequencing was performed using Illumina Miseq (San Diego, CA, United States). The library was created by 35-cycle PCR with primers Pro341F 5′-CCTACGGGNBGCASCAG-3′ (Takahashi et al., 2014) and Pro805R 5′-GACTACNVGGGTATCTAATCC-3′ (Herlemann et al., 2011). To ensure the quality of the sequencing run and analysis, a sequencing mock community (ZymoBIOMICS™ Microbial Community Standard, Zymo Research Corp., Irvine, CA, United States, Cat. No. D6300) was included in the sequencing project. The sequence data were analyzed using QIIME2 (Bolyen et al., 2019) and DADA2 (Callahan et al., 2016). All sequencing raw data are available in the NCBI Sequence Read Archive (SRA^3^) with the BioProject ID: PRJNA745084^4^.

## Results

The variations caused by fosfomycin on WWC structures were monitored using a bioreactor setup operated in a continuous mode. The dynamics of structure changes were measured on the single cell level using flow cytometry. Sorted subsets of cells were further investigated by 16S rRNA amplicon sequencing to unravel species that were persistent under the pressure of fosfomycin. In addition, we used a metabolically versatile *P. putida* strain as a model organism to track the fate of a typical member of a complex WWC, which could also be implemented as a potential future chassis for fosfomycin degradation pathways. The fate of the *gfp*-labeled derivative of *P. putida* KT2440 was tracked both in fosfomycin undisturbed batch cultivations and under fosfomycin disturbed continuous-mode bioreactor operation.

### Dynamics of Batch-Grown Undisturbed WWCs

**FIGURE 1:**
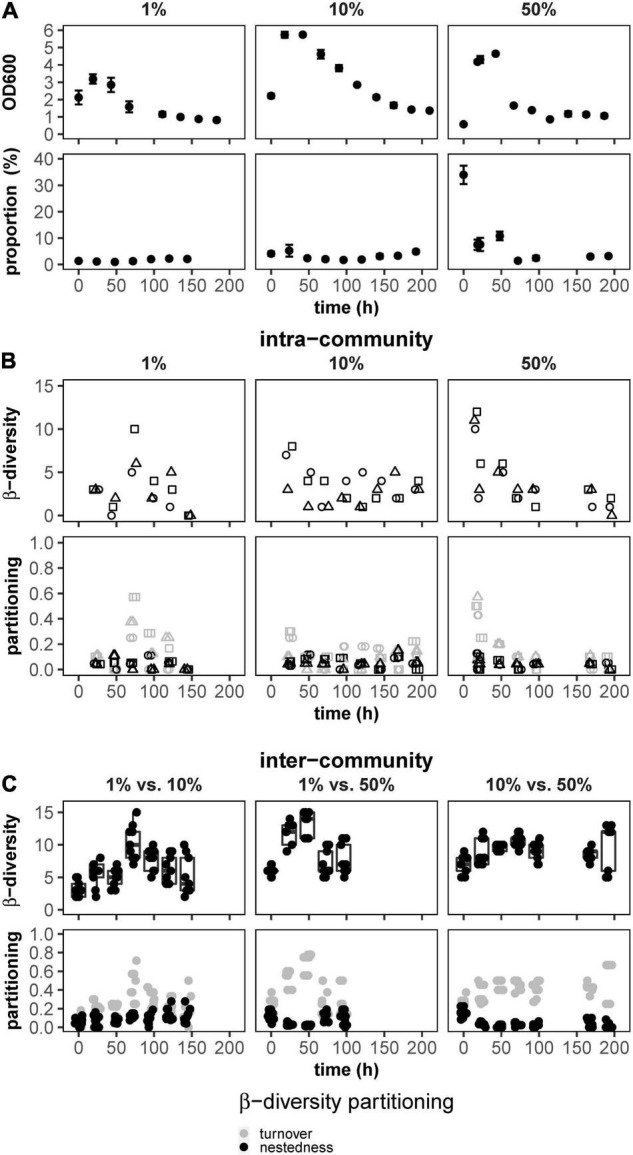
Growth and ecological characteristics of nine batch-cultured WWC co-cultures augmented by proportions of *gfp*-labeled *P. putida* KT2440 (1, 10, and 50%; left to right). **(A)** Top: OD changes over 192 h, **(A)** bottom: changes of proportions of *gfp*-labeled *P. putida* KT2440 within the WWCs. **(B)** Top: variation in intracommunity β-diversity values between successive time points over 192 h. **(B)** Bottom: partitioning of intracommunity β-diversity into turnover and nestedness components between successive time points over 192 h. **(C)** Top: intercommunity β-diversity between pairs of WWCs per co-culture setup. **(C)** Bottom: partitioning of intercommunity β-diversity into turnover and nestedness components over 192 h. All cultivations were done in triplicates.

The *gfp*-labeled derivative of *P. putida* KT2440 was bio-augmented in different proportions (1, 10, and 50%, see section “Materials and Methods”) into triplicate batch-grown WWCs. For all nine setups, the OD reached maxima between 24 and 48 h and declined to an OD of about 1 at 192 h (Figure 1A, top) due to stalled cell growth and cell death common for stationary phases of batch cultivations. The green fluorescence cells of the *gfp*-labeled *P. putida* KT2440 were also DAPI-stained and thus part of the DAPI vs. FSC community fingerprint. The position of the *gfp*-labeled *P. putida* KT2440 in the DAPI vs. FSC community fingerprint was marked by a gate, and the proportions of the green fluorescent cells compared to the cells of the WWC were determined (Supplementary Figure 4.1). At all three augmented proportions of *gfp*-labeled *P. putida* KT2440 cells declined rather fast (Figure 1A, bottom), which was also visualized for the FSC vs. greenFI 2D-plots in the movies S2.1 (1%), S2.2 (10%), and S2.3 (50%) and for the FSC vs. DAPI 2D-plots in the movies S2.4 (1%), S2.5 (10%), and S2.6 (50%) indicating no growth, out-competition, or even death of the augmented strain. To visualize WWC structural trends over 192 h for all nine community setups, we calculated intracommunity β-diversity highlighting the differences between successive samples per WWC. In all nine WWCs, the differences subsided over time (Figure 1B, top). These data indicate that in a batch culture, as time progressed, fewer and fewer cell types were able to grow in addition to those that already dominated the communities because further growth was not possible due to nutrient limitation. In addition, intracommunity β-diversity partitioning (β_SOR_, Baselga, 2010; Liu and Müller, 2020) suggested that turnover decreased in intracommunity β-diversity after 192-h cultivation, while nestedness remained low and unchanged (Figure 1B, bottom). Comparisons were also made between two WWCs of a triplicate, and this was done for all three co-cultures, taken at the same time points and over 192 h, respectively. These calculations showed that intercommunity β-diversity remained high over all nine WWC setups (Figure 1C, top), while the number of nested subcommunities that were present in all nine communities was low (Figure 1C, bottom).

Therefore, although the variation within each of the nine WWCs was minimized until the stationary phase of growth, distinctly different community compositions were seen between the nine WWCs, while all, however, did not support the augmented *gfp*-labeled *P. putida* KT2440.

### Dynamics of a Continuously Grown WWC Under Fosfomycin Pressure

**FIGURE 2:**
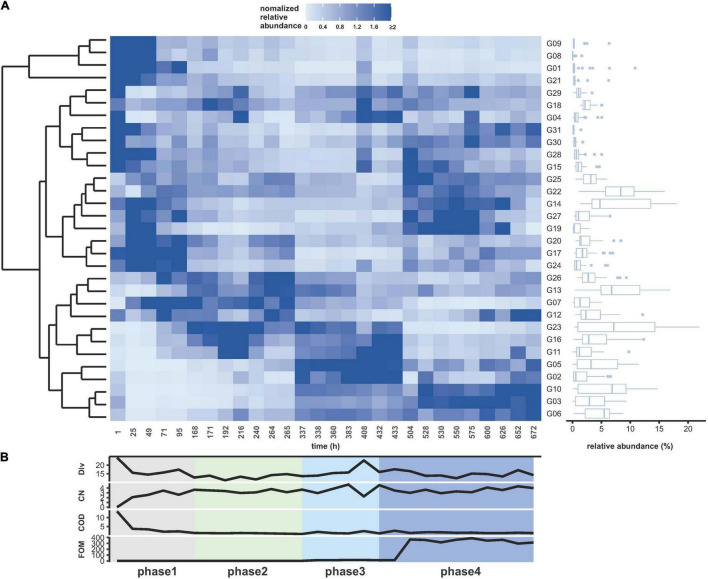
Community dynamics during continuous cultivation for 672 h. **(A)** Cytometrically analyzed relative cell abundances (%) per subcommunity relative to the number of cells for each measurement (G01–G31). The relative cell abundance values were normalized by the average relative cell abundance per subcommunity over time (blue: higher than average, light blue: lower than average). The relative cell abundance per subcommunity was summarized as box plot (right side). The upper and lower edges of the boxes indicate 75th and 25th percentile; the middle line indicates the median. Points indicate the outliers. Clustering of subcommunities was arranged according to the similarity of their abundance changes over time (left side). **(B)** Variation in the concentrations of abiotic parameters from bottom to top: FOM (fosfomycin, mg L^–1^), COD (O_2,_ mg ml^–1^), and CN (×10^10^ counts ml^–1^). In addition, the variation in α-diversity (Div, order-based Hill number *q* = 2) was given. The experiment was subgrouped in four phases—gray, adaptation; green, gfp-labeled *P. putida* KT2440 augmentation; light blue, fosfomycin 10 mg L^–1^; blue, fosfomycin 268 mg L^–1^ (details in Table 1).

A WWC was grown in a continuous bioreactor setup over a period of 672 h. With the exception of oscillating dissolved oxygen concentration, other abiotic bulk parameters such as aeration, pH, temperature, and stirring velocity were measured and controlled online and found largely unchanging (Supplementary Figure 3.1). Biotic bulk parameters were measured offline such as OD, CN, and COD and remained also mostly on the same level after an adaptation period (Supplementary Table 3.1). Fosfomycin levels were always measured slightly higher than the added concentrations, and no degradation activities were observed (Figure 2B).

Flow cytometric FSC vs. DAPI parameter measurement of 28 samples has been providing a data set of 868 subcommunities on the basis of 31 defined gates per sample. The experiment was divided into four phases with phase 1 comprising the adaptation time of the WWC to the continuous reactor conditions, phase 2 with the start of the addition of the *gfp*-labeled *P. putida* KT2440, phase 3 with the addition of 10 mg L^–1^ fosfomycin, and phase 4 with the addition of 268 mg L^–1^ fosfomycin. The different phases were color-coded, and the bio-augmented *gfp*-labeled *P. putida* KT2440 was added at proportions of 0.5–2.5% of the WWC (Table 1, Supplementary Figure 4.3, and Supplementary Movies 4.1, 4.2). Using dissimilarity analysis, the cultivated pure *gfp*-labeled *P. putida* KT2440 strain was found clearly separated from the continuously cultivated WWC (Supplementary Figure 5.1).

The changes in the community structure (subcommunities G1–G31) are shown in Figure 2A with the color key indicating increases in relative cell abundance per subcommunity by increasingly darker blue shades. After the initial adaptation to the continuous reactor conditions, the composition of the community changed constantly, which continued in phase 2 to phase 4. The gradual structure shift in phase 4 seems to have been influenced by the addition of 268 mg L^–1^ fosfomycin. The most dominant subcommunities during the whole experiment were G13–G14 and G22–G23, while the most markedly increasing subcommunities were G03, G06, and G10 after the addition of high concentrations of fosfomycin in phase 4.

**FIGURE 3:**
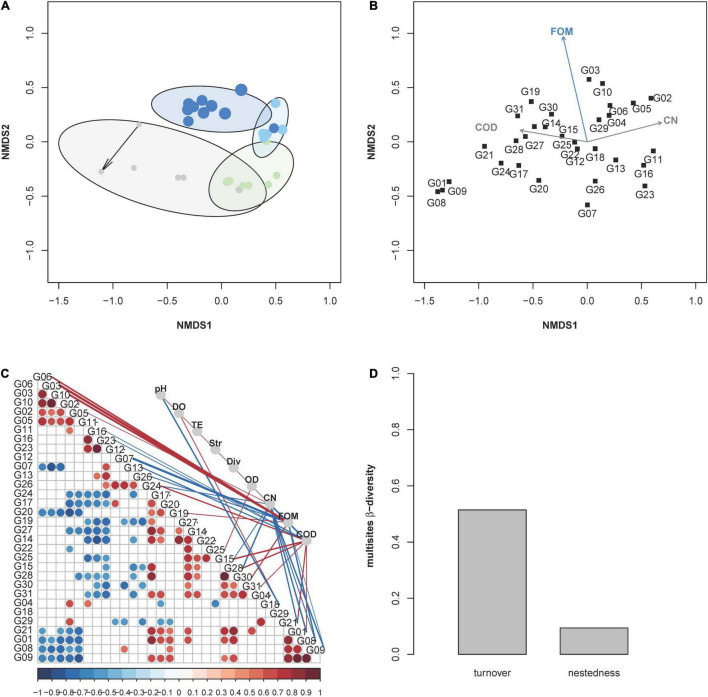
Trends in community evolution depending on fosfomycin concentrations. **(A)** The assembly process of the microbial community in the bioreactor was visualized *via* a NMDS plot based on the dissimilarity analysis (Bray–Curtis index) of relative cell abundance per subcommunity. Each point in the NMDS plot represent a whole community sample, and the increase in its sampling time was represented by the increased point size. Points were colored according to the sampling phases, and the starting point was marked by an arrow. The experiment was subgrouped in four phases—gray, adaptation; green, gfp-labeled *P. putida* KT2440 augmentation; light blue, fosfomycin 10 mg L^–1^; blue, fosfomycin 268 mg L^–1^ (details in Table 1). **(B)** Relationship between abiotic parameters and abundance variations of subcommunities (R function “envfit” from the vegan package); only significant correlations of | *rho*| > 0.7 and *p*-value < 0.05 were displayed such as FOM, CN, and COD. **(C)** Relationship between subcommunities and environmental parameters. The correlation was performed using Spearman rank index *rho*. Only significant correlations | *rho*| > 0.5 and *p*-value < 0.05 were displayed. The strengths of the correlation values were indicated by the size of points (subcommunities vs. subcommunities) or the thickness of the edges (subcommunities vs. environmental parameters) and by the color code below the *x*-axis (red: positive, blue: negative). Explanation of abbreviations: pH, DO (dissolved oxygen concentration), T (temperature), Str (stirring), Div (order-based Hill number *q* = 2), OD, CN (cell number), FOM (fosfomycin, mg L^–1^), and COD (O_2_ mg ml^–1^). **(D)** Multisite intracommunity β-diversity from 432 to 672 h using the R function “betapart.” Turnover and nestedness values within the whole community during continuous cultivation.

The trend in community evolution during the four phases was visualized by a NMDS plot. The difference in intracommunity composition within phases was equalized from phase 1 to phases 3 and 4. In contrast, successive changes were found to occur between phases as responses to the addition of fosfomycin (Figure 3A and Supplementary Movie 4.2). The responsible subcommunities for these developments were found by correlation analysis, which indicated growing subcommunities by increases in CNs (e.g., G05 and G02) or subcommunities that correlated their fate with COD values (e.g., G31 and G15), or lost (e.g., G07 and G26) or profited (e.g., G3, G06, and G10) by increases in fosfomycin (Figure 3B). Therefore, increasing FOMs partially drove the community change from phase 2 to phase 4. The correlations were performed based on relative cell abundances of subcommunities using Spearman’s correlation index *rho*, and only strong I*rho*I > 0.7 was shown in Figure 3B. The *p*-value per correlation was adjusted according to Benjamini and Hochberg (1995). Specifically, fosfomycin showed negative correlations with subcommunities (e.g., G08 and G09), and these, in turn, showed negative correlations with CNs, indicating a loss of subcommunities due to fosfomycin treatment (Figure 3C). The fosfomycin-supported subcommunities also reinforced each other (e.g., G3, G6, and G10) and, in addition, showed negative correlations to fosfomycin-inhibited subcommunities (G08 and G09). Not influenced by fosfomycin were subcommunities of, e.g., G11, G16, and G23, which showed a negative relationship to ten other subcommunities and COD (Figure 3C) and were also visualized separate from all other subcommunities in Figure 3B. *P. putida* was found in gates G15, G30, and G31 and shown to be positively correlated to fosfomycin and COD; however, the strain only accounted for less than 50% of cells in these gates, and neither gates had been dominant in the community.

To verify if some of the dominant subcommunities (relative abundance >3.23%) remained persistent under high fosfomycin pressure (e.g., fosfomycin-resistant bacterial genera), a multisite intracommunity β-diversity comparison was made to unravel nested subcommunities. For this, 341 subcommunities of the 11 communities during phase 4 were tested for persistence (Supplementary Figure 5.2). The nestedness value was very low with 0.09 in comparison to the turnover value of 0.51 but revealed the subcommunities G03, G06, G10, G13, G14, and G22 as those that persisted in the bioreactor during phase 4 (Figure 3D and Supplementary Figure 5.2). The subcommunities G13, G14, and G22 were dominant through all phases, while the subcommunities G03, G06, and G10 only dominated and persisted in phase 4. The high turnover value supported the findings in Figure 2 where massive community shifts were observed along the phases.

### 16S rRNA Analysis of Selected Whole Communities and Sorted Subcommunities

The taxonomic composition of the wastewater community was determined before and after the impact of high FOM of 268 mg L^–1^. For this, samples were taken from phase 1 at 1 h and phase 4 at 432 h (starting point phase 4) and at 505 h. From the sample 505 h three gates persistent under 268 mg L^–1^ FOM s were sorted (Supplementary Figure 5.2). The subcommunity G10 was abundant at averaged values (5.95 ± 4.93%) and was strongly supported by fosfomycin. The subcommunities G14 and G22 were among the most abundant ones during all phases of the continuous cultivation (average value 8.08 ± 5.58 and 8.61 ± 3.78%, respectively) and were the major subcommunities composing the endpoint community after high fosfomycin pressure (Supplementary Figure 5.2).

**FIGURE 4:**
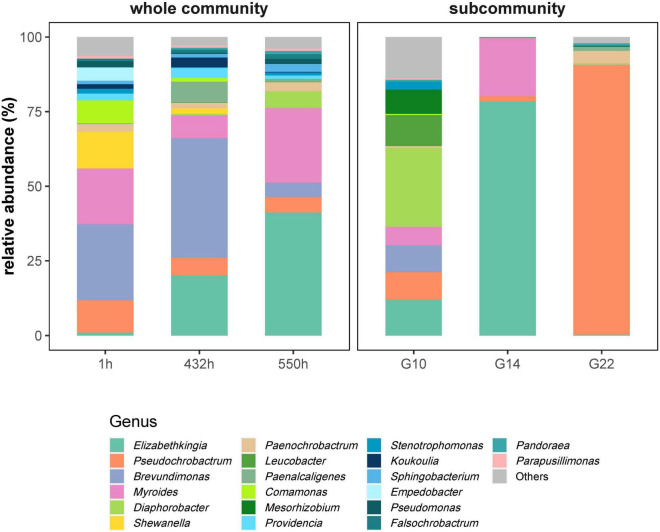
The relative abundances (%) of genera in whole communities and sorted subcommunities. Whole communities at 1 h (starting time point of phase 1: adaptation), 432 h (starting time point of phase 4: 268 mg L^–1^ fosfomycin), and 550 h (a middle time point of phase 4: 268 mg L^–1^ fosfomycin). Subcommunities G10, G14, and G22 sorted from 550-h community. G10, G14, and G22 were persistent over phase 4.

The whole community showed a successive increase in the genera *Elizabethkingia* from nearly 0 to 41.19% at 1 h and *Myroides* from 7.73 to 25.08% at 550 h (Figure 4, left). The genus *Brevundimonas* was obviously inhibited by 268 mg L^–1^ fosfomycin from 40.01% at 432 h to 4.8% at 550 h. The genera *Diaphorobacter* (5.59%) and *Pseudochrobactrum* (5.22%) represented persistent members but with lower abundance at 550 h (Figure 4, left).

Sorted cells from persistent subcommunities G10, G14, and G22 under high fosfomycin pressure revealed 90.28% of the genus *Pseudochrobactrum* in G22 as well as 78.47 and 19.47% of the genera *Elizabethkingia* and *Myroides* in G14. G10 showed a multigenera composition with 26.48% of the genus *Diaphorobacter*, while all of the previously mentioned genera and additionally the genera *Leucobacter* and *Mesorhizobium* were represented with about 5% (Figure 4, right).

## Discussion

The objective of this work was to investigate the long-term impact of low (10 mg L^–1^) and high (½ EC 50, 268 mg L^–1^) concentrations of the re-emergent fosfomycin on a microbial wastewater community (WWC) and to unravel if the antibiotic is disrupting the composition of the community and selects for specific microorganism. In addition, the survival capacity of the added *gfp*-labeled *P. putida* KT2440 within the community and under fosfomycin pressure was tested.

In a recent study, we have observed that generally undisturbed municipal WWTPs strongly sustain different but functionally active community compositions in nearly all basins and reactors over long time periods (Vucic et al., 2021). Contrarily, we found that communities cultivated in parallel insular bioreactor environments trajected into different compositions (Liu et al., 2019). A similar behavior was observed in our nine parallel but independently conducted batch experiments where the communities also trajected into different directions and were dissimilar to each other. However, typical nutrient limitation in batch cultivation prevented further changes in community composition (no turnover), and low intracommunity β-diversity indicated the stalling of community growth. Thus, referring on batch cultivation adopts an artificial view on community behavior and does not reflect community dynamics and responses to disturbance events occurring in managed or environmental settings.

Therefore, we measured the dynamics of the wastewater community under continuous cultivation conditions inclusive of the addition of the antibiotic fosfomycin at low and high concentrations. The cytometric diversity parameters did not unravel the action of fosfomycin: the α-diversity (order-based Hill number *q* = 2) and intracommunity β-diversity values (Supplementary Figure 5.2) were not effective in detecting specific community trends. Also, noteworthy variations in biomass production measured by OD and CNs, or in COD, were not indicated (Figure 2). Regardless, the community composition changed. The community trajectory suggested that microbial communities tolerated low concentrations of fosfomycin, although the study period chosen may have been too short to find clearer traces of slow changes (Figure 3A). Instead, high concentrations of fosfomycin caused a turnover of cytometric community structure, reshaped the community, and simultaneously selected for persistent subcommunities (Figure 3). These findings were supported by exemplary 16S rRNA gene sequencing, which showed a compositional shift of the WWC where, e.g., the genus *Brevundimonas* was lost and the genera *Elizabethkingia* and *Myroides* increased in relative abundance. Therefore, the proportions of cell types changed and with this the community composition, while CN, OD, or COD values remained unchanged.

We showed by correlation analysis that specific cytometric subcommunities were functionally interactive with and supported by fosfomycin, while others were obviously inhibited by the antibiotic as their CN went down (subcommunities G08 and G09). Therefore, the change in the proportions of the subcommunities in the community demonstrated the selection of cell types. Some of the subcommunities that survived even the high FOM were sorted for 16S rRNA analysis. Tolerance to these high FOM s could be supported by specific characteristics of strains belonging to the dominant genera found under fosfomycin pressure. One of those was the highly abundant genus *Pseudochrobactrum* in the subcommunity G22. These bacteria are well known for their heavy metal resistance, e.g., a *Pseudochrobactrum saccharolyticum* strain has been described to be involved in the efficient removal of Cr(VI) (Li et al., 2019). Heavy metal resistance has been shown to frequently co-select with antimicrobial resistance, so increased abundance of this genus under the given conditions is not surprising (Dickinson et al., 2019). Cell abundance of the genus *Myroides* was also increasing under fosfomycin pressure, and their members have been described to be highly resistant to most available antibiotics (Hu et al., 2016); among the genus *Elizabethkingia*, clinical isolates have been found that were containing different lactamase- and efflux protein-encoding genes (Chen et al., 2019). Therefore, although Bottery et al. (2021) argued that the effect of antibiotics on bacteria is slowed by interspecies relationships in highly diverse communities through mechanisms such as collective resistance, collective tolerance, and exposure protection, the continuous reactor setup strongly selected for genera in the WWC that are well described to cover the species involved in pathogenesis and antibiotic resistance. These bacteria were obviously able to sustain the action of fosfomycin under continuous reactor conditions, and they covered nearly 70% of the whole community after 550 h of cultivation.

It is obvious that measures must be undertaken to avoid the spread of antibiotic-resistant species. Some earlier studies already adopted biological processes to treat pharmaceutical wastewater. McGrath et al. (1998) isolated *Rhizobium huakuii* with fosfomycin degradation capabilities. This strain belongs to the genus *Mesorhizobium*, which was also found in our community and even to above 8% in the subcommunity G10. We, therefore, tested in our study how well a bio-augmented *P. putida* strain might survive. While having inherent resistance capacities to fosfomycin (Falagas et al., 2019) but no degradation capabilities, this strain was, under all conditions, outcompeted. This highlights the difficulties of sustainment of augmented functionally valuable strains in natural microbial communities over long periods of time. Nevertheless, *P. putida* KT2440 is a genetically well-studied organism, often used for genetic engineering in synthetic biology (Nikel et al., 2014) and may contribute to a solution in the future, although this would require closed-system wastewater treatment facilities.

The use of high-throughput flow cytometry, cell sorting, and selected 16S rRNA gene sequencing in combination with continuous cultivation of a WWC allows for assessing the impact of antibiotics on natural or managed microbial communities in general and on individual community members in particular. This inexpensive and rapid technology is a more in-depth approach than classical cultivation techniques and is also likely to be less labor intensive compared to single-cell Raman spectroscopy or metagenomics in visualizing changes in community structure in response to antibiotic pressure. In particular, the usefulness of flow cytometry to analyze the effects of low fosfomycin pressure over long periods of time may be of general interest, as this is the more typical pressure that WWCs must deal with. However, preventing or minimizing the release of antibiotics such as fosfomycin into WWTPs by manufacturers would be the most promising step in reducing the further spread of fosfomycin resistance.

## Data Availability Statement

The datasets presented in this study can be found in online repositories. The names of the repository/repositories and accession number(s) can be found below: NCBI (accession: PRJNA745084) and FlowRepository (repository ID: FR-FCM-Z46E).

## Author Contributions

SL performed the bioreactor experiment, evaluated the data, and wrote the manuscript. ZL measured and evaluated the cytometric data and revised the manuscript. CS performed the bioreactor experiment. LC measured the fosfomycin concentration. SM designed the experiment and wrote the manuscript. PZ performed the batch experiments and revised the manuscript. All authors contributed to the article and approved the submitted version.

## Conflict of Interest

The authors declare that the research was conducted in the absence of any commercial or financial relationships that could be construed as a potential conflict of interest.

## Publisher’s Note

All claims expressed in this article are solely those of the authors and do not necessarily represent those of their affiliated organizations, or those of the publisher, the editors and the reviewers. Any product that may be evaluated in this article, or claim that may be made by its manufacturer, is not guaranteed or endorsed by the publisher.

## Footnotes

1
https://flowrepository.org/id/FR-FCM-Z46E

2
https://www.bioconductor.org/packages/release/bioc/html/flowCyBar.html

3
https://www.ncbi.nlm.nih.gov/sra

4
http://www.ncbi.nlm.nih.gov/bioproject/745084
