# Supplementary material for: The Impact of the Antibiotic Fosfomycin on Wastewater Communities Measured by Flow Cytometry
Source: Front Microbiol. 2022 Mar 3;12:737831. doi: 10.3389/fmicb.2021.737831 (PMC8928225; doi:10.3389/fmicb.2021.737831)
Supplement: Supplementary file 9 [file Data_Sheet_1.PDF]

Supplementary Information

The impact of fosfomycin on wastewater communities measured by flow cytometry

Shuang Li[1], Zishu Liu[2], Christine Süring[1], Luyao Chen[3], Susann Müller[1], Ping Zeng[3]

[1] Helmholtz Centre for Environmental Research – UFZ, Department Environmental Microbiology Permoserstraße 15 / D-04318 Leipzig, Germany
[2] Zhejiang University, College of Environmental and Resource Sciences, Hangzhou 310058, China
[3] Institute of Water Ecology and Environment, Chinese Research Academy of Environmental Sciences, Beijing 100012, China

Content

S1: Batch growth of *gfp*-labeled *P. putida* KT2440
S2: Batch-cultivated wastewater communities (WWC)
S3: Wastewater communities cultivated under continuous reactor environments
S4: Cytometric methods
S5. Diversity calculations

S1: Batch growth of *gfp*-labeled *P. putida* KT2440

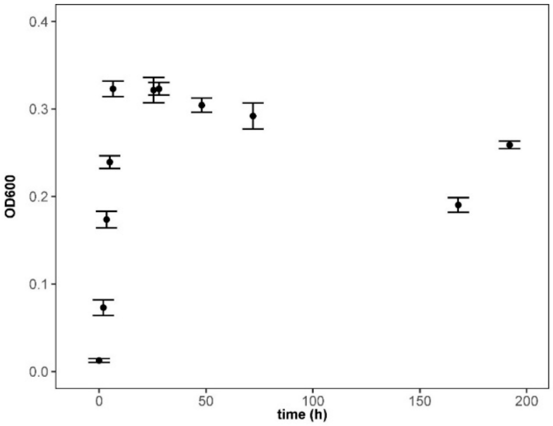

Figure S1.1: Growth curve of gfp-labeled *P. putida* KT2440 in 100 mL LB (Lysogeny broth) medium in 500 mL flasks at 30 °C, 125 rpm and for 192 h (n=3).

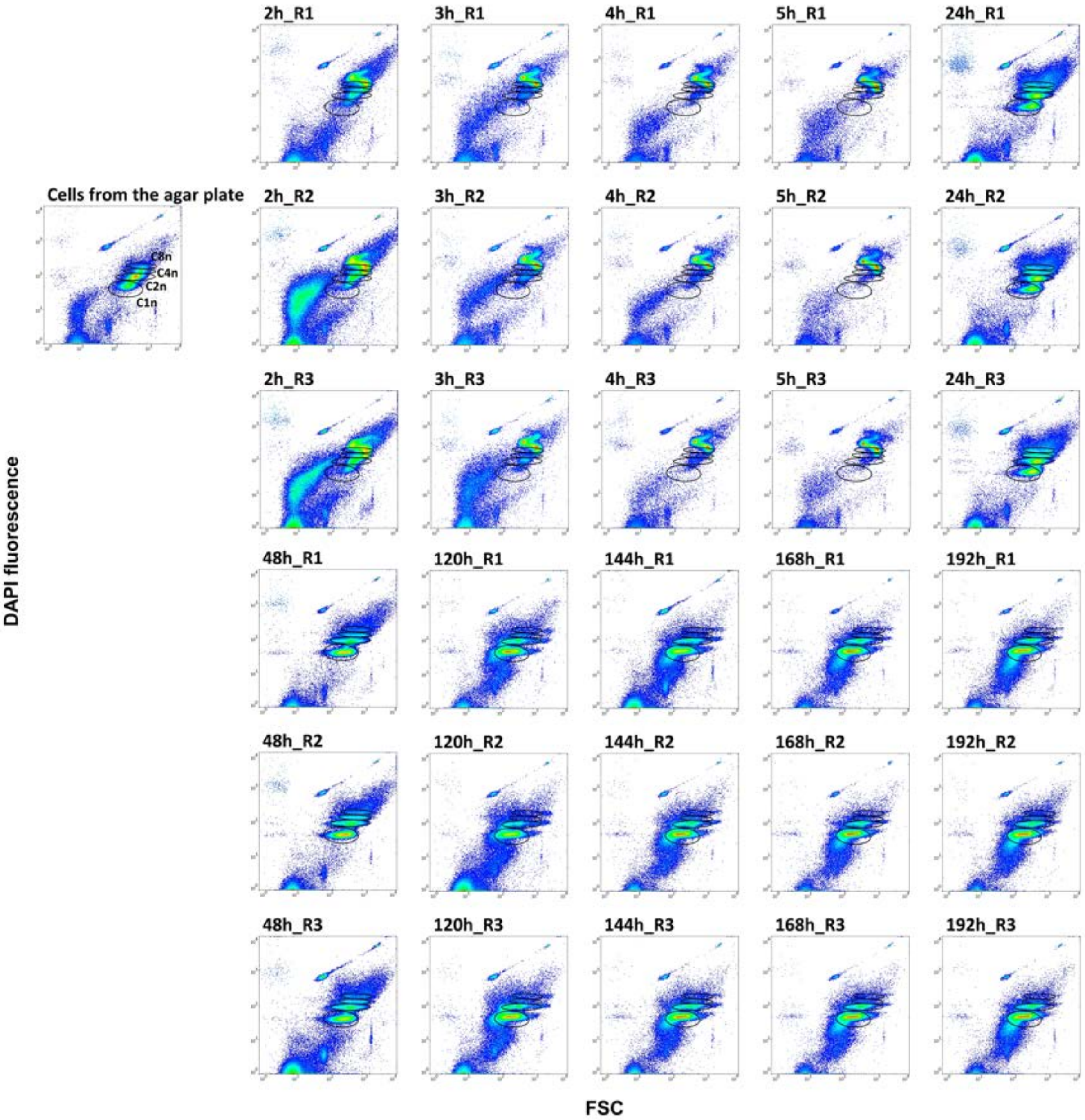

Figure S1.2: DAPI vs. FSC 2D-plot growth pattern of gfp-labeled *P. putida* KT2440 measured by flow cytometry (DAPI: blue fluorescence, measurement of DNA, FSC: measurement related to cell size). The growth was followed for 192 h. For the inoculum, a colony was taken from an LB agar plate after 72h of growth (left above). Exponential growth was analyzed until 5h, afterwards the population segregated into four and finally, after 120 h, into 2 subpopulations with cells containing lower chromosome numbers. The 4 gates located from bottom to top on each plot marked populations of C1n, C2n, C4n, C8n. (R1-R3: replicates).

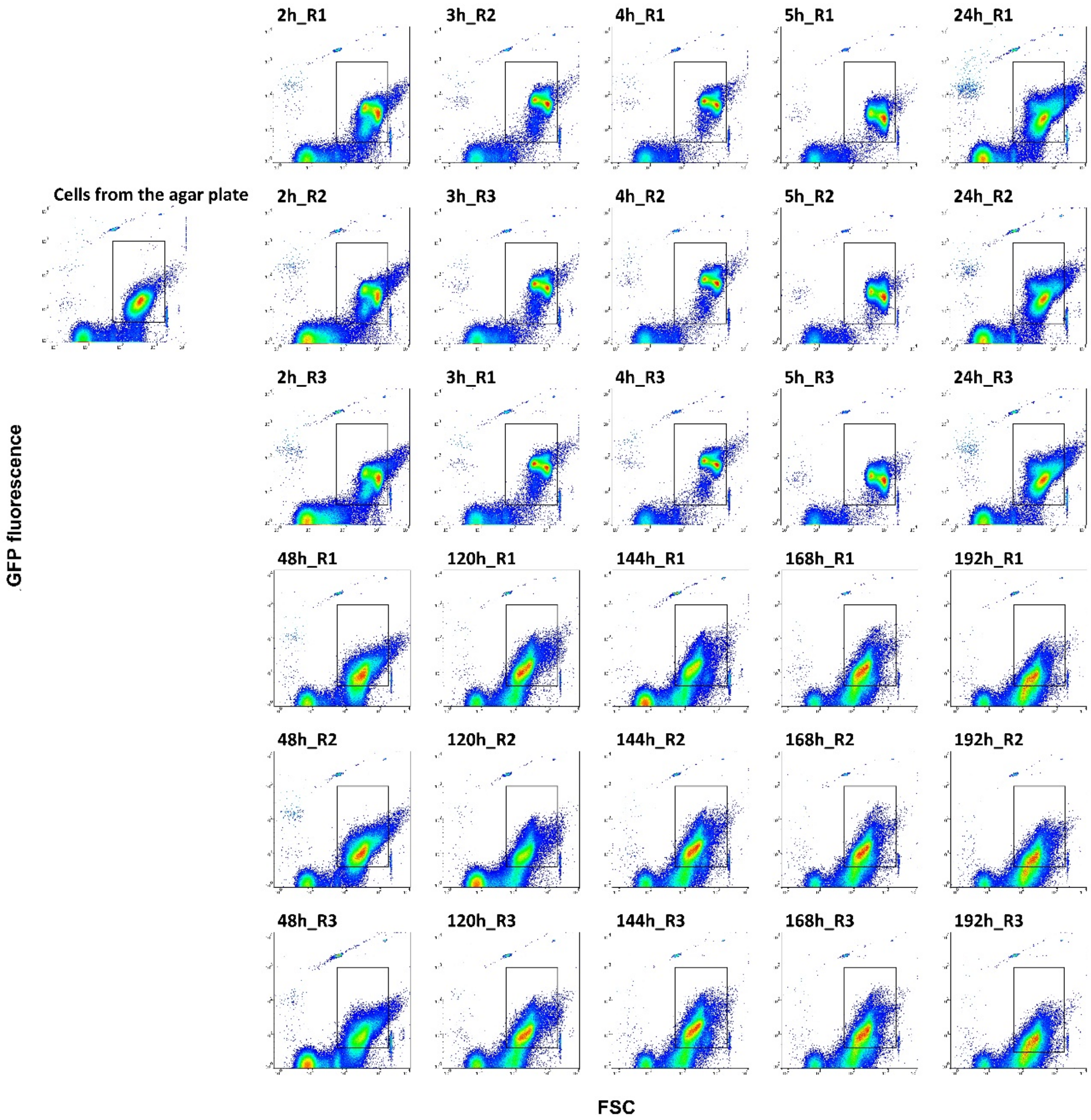

Figure S1.3: GFP-autofluorescence variations of gfp-labeled *P. putida* KT2440 measured by flow cytometry (green fluorescence: GFP, FSC: measurement related to cell size). The growth was followed for 192 h. For the inoculum, a colony was taken from an LB agar plate after 72h of growth (left above). The upper right quadrant was used for counting GFP-autofluorescent cells. (R1-R3: replicates).

S2: Batch-cultivated wastewater communities (WWC)

The batch cultivations were performed in 100 mL LB medium in 500 mL flasks at 30 °C, 125 rpm for 192 h in triplicates. WWC and gfp-labeled *P. putida* KT2440 were mixed in following respective proportions: 99:1, 9:1, 1:1 (prepared via OD). DAPI vs. FSC 2D-plot growth patterns of gfp-labeled *P. putida* KT2440 were measured by flow cytometry by UV excitation (355 nm). GFP-autofluorescence variations were measured by blue excitation (488 nm). The inoculum originated from the activated sludge basin of a nearby WWTP. For GFP-autofluorescence, the upper right quadrant was used for counting GFP-autofluorescent cells (Figure S1.3). For DAPI vs. FSC 2D-plot growth patterns a gate template based on the cell gate was created (Figure S4.1). The changes in community patterns were analyzed as 2D-plots and visualized as movies for the DAPI vs. FSC 2D-plot growth pattern and the fluorescence intensity of the GFP-autofluorescence of the *P. putida* strain:

Movies S2.1 1-gfp-fsc (1 %), S2.2 10-gfp-fsc (10 %) and S2.3 50-gfp-fsc (50 %)
Movies S2.4 1-dapi-fsc (1 %), S2.5 10-dapi-fsc (10 %), and S2.6 50-dapi-fsc (50 %)

S3: Wastewater communities cultivated under continuous reactor environments

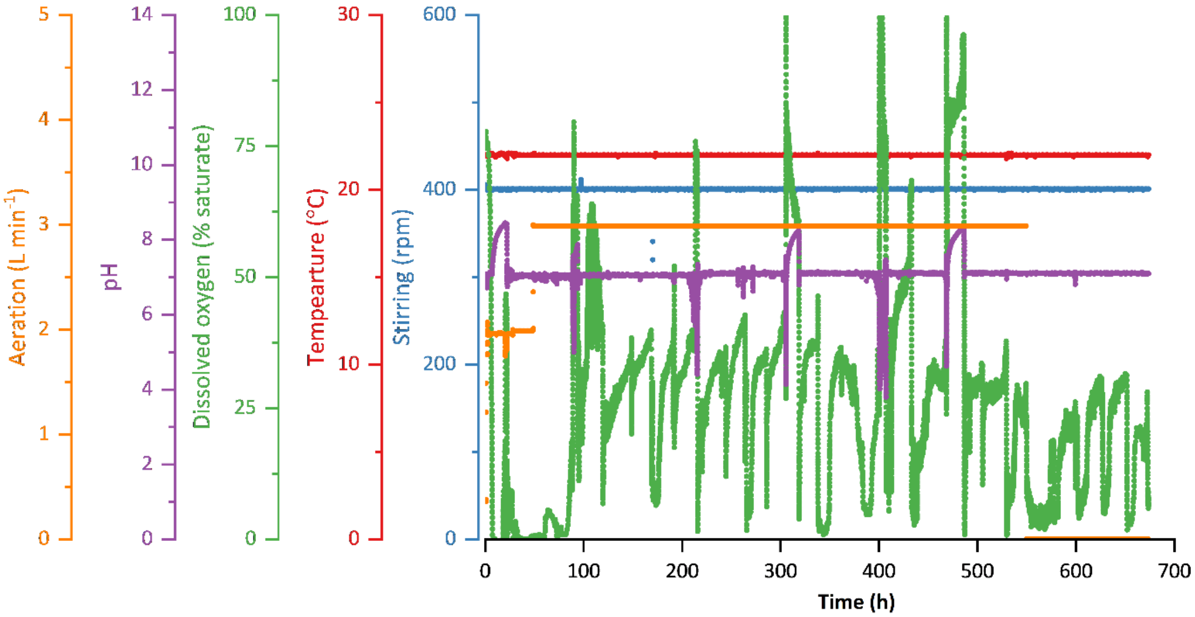

Figure S3.1: Continuous cultivation of the microbial community in the bioreactor (see Materials and Methods). Overview on abiotic data, measured on-line over a time range of 672 h. While the dissolved oxygen concentration oscillated, also caused by small fluctuations in the aeration rate and pH control, temperature and stirring values remained constant.

Off-line measured values for optical density (OD), cell number (CN), fosfomycin (FOM) and COD values are listed in Table S3.1.

Table S3.1: Optical density (OD), cell number (CN), fosfomycin (FOM), COD values

Group	Sample No.	Time (h)	$OD_{600nm,\lambda=5mm}$		Cells numbers ($\times 10^{10}$ cells/mL)		FOM (mg/L)		COD (g/L)	
			mean	±SD	mean	±SD	mean	±SD	mean	±SD
Adap-tation	0	1	0.0843	0.0015	0.021	0.002	*0.0		13.40	1.25
	1	25	0.5453	0.0119	2.099	0.095	*0.0		3.70	0.09
	2	49	0.4980	0.0010	2.560	0.144	*0.0		3.42	0.19
	3	71	0.5580	0.0095	3.464	0.007	*0.0		2.12	0.10
	4	95	0.4503	0.0081	2.594	0.036	*0.0		2.28	0.03
Addition of KT2440	5	168	0.5107	0.0076	3.635	0.090	*0.0		1.41	0.08
	6	171	0.4993	0.0091						
	7	192	0.5097	0.0051	3.370	0.018	*0.0		1.22	0.07
	8	216	0.4607	0.0162	2.962	0.053	*0.0		1.39	0.02
	9	240	0.4807	0.0045	3.083	0.282	*0.0		1.29	0.09
	10	264	0.5093	0.0055	3.778	0.174	*0.0		1.14	0.14
	11	265	0.5237	0.0015	3.119	0.267				
FOM10 + addition of KT2440	12	337	0.5243	0.0276	3.664	0.025	*0.0		0.83	0.03
	13	338	0.5133	0.0083	2.938	0.045	11.20	1.70	2.02	0.03
	14	360	0.5513	0.0086	3.828	0.666	10.90	2.65	1.32	0.11
	15	383	0.5213	0.0093	4.716	0.326	15.00	2.21	1.15	0.18
	16	408	0.4377	0.0081	2.247	0.190	14.00	2.00	2.45	0.23
FOM268 + addition of KT2440	17	432	0.4377	0.0081	4.585	0.157	10.20	1.98	1.17	0.16
	18	433	0.4813	0.0025	3.466	0.050	12.00	1.65	2.65	0.05
	19	504	0.5740	0.0020	2.990	0.226	366.63	35.23	1.40	0.14
	20	528	0.5793	0.0049	3.648	0.147	357.03	11.49	1.81	0.52
	21	530	0.5447	0.0085	2.911	0.179	316.17	8.83	1.79	0.43
	22	550	0.5843	0.0071	3.291	0.089	362.47	52.74	1.49	0.22
	23	575	0.4997	0.0136	3.107	0.336	388.23	4.53	1.58	0.13
	24	600	0.5567	0.0021	4.095	0.183	346.73	2.80	1.32	0.08
	25	627	0.5537	0.0162	3.569	0.101	361.27	22.37	1.33	0.09
	26	652	0.5860	0.0066	4.355	0.225	296.20	3.96	1.54	0.16
	27	672	0.5220	0.0092	3.969	0.025	314.97	3.25	1.36	0.17

*observed FOM concentration lower than the lowest detecting limit, thus set as zero

The experiment was sub-grouped into for four phases: grey: adaptation, green: *gfp*-labeled *P. putida* KT2440 augmentation, light blue: fosfomycin 10 mg L^{-1}, blue: fosfomycin 268 mg L^{-1}**.**

S4: Cytometric methods

For the evaluation of cytometric data, first a cell gate for measuring 200,000 cells is defined in addition to the gate template, which allows to determine relative cell abundance variation per subcommunity over time. For the samples from the batch cultivations and the continuous reactor different cell gates and gate templates were created.

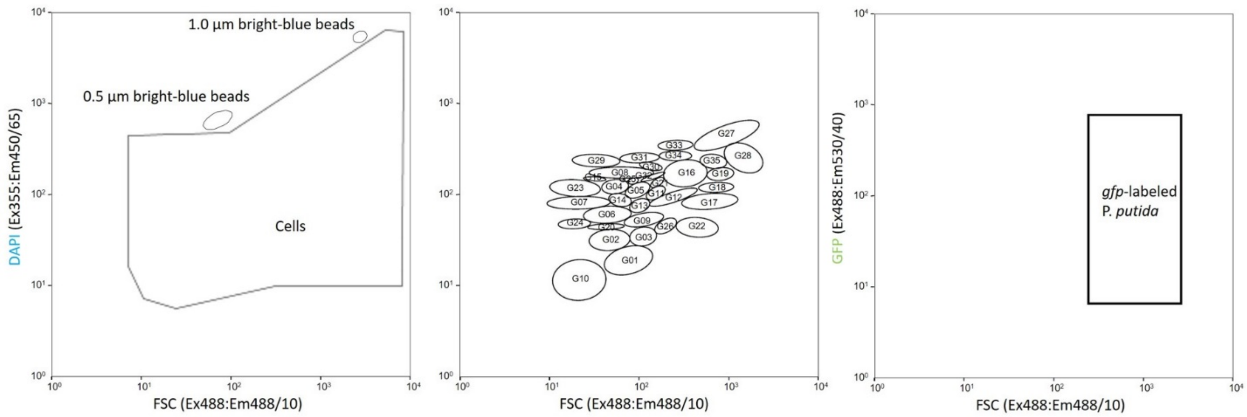

Figure S4.1: Cell gate (left) and gate template (center) for the evaluation of DAPI vs. FSC 2D-plots for the batch co-culture of the WWC and the *gfp*-labeled *P. putida* KT2440. The *gfp*-labeled P. *putida* gate (right) marks the green fluorescent cells in the GFP vs. FSC 2D-plots. Calibration beads and noise were excluded from the analysis.

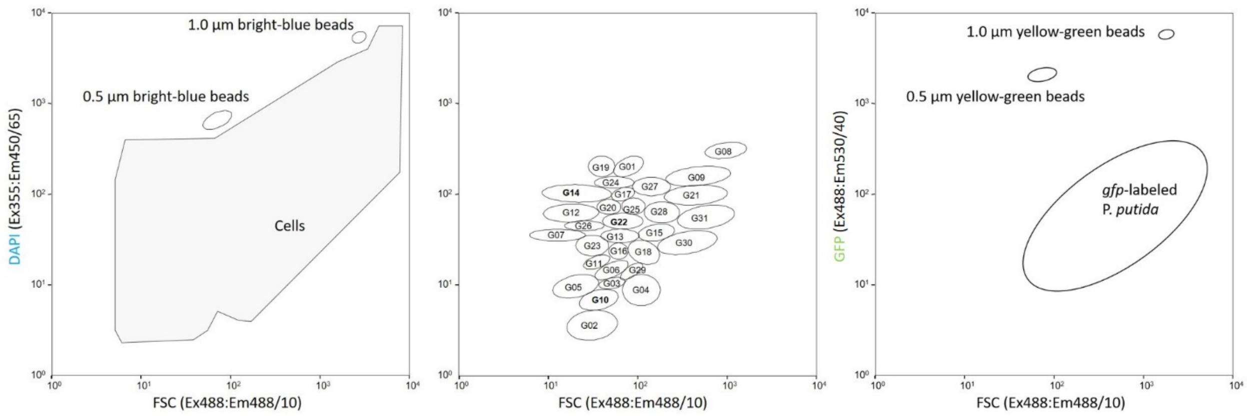

Figure S4.2**:** Cell gate (left) and gate template (center) for the evaluation of DAPI vs. FSC 2D-plots for the continuous cultivation of the WWC and the *gfp*-labeled *P. putida* KT2440. The *gfp*-labeled P. *putida* gate (right) marks the green fluorescent cells in the GFP vs. FSC 2D-plots. Calibration beads and noise were excluded from the analysis. In addition, the position of the sorted subcommunities (G10, G14, and G22) in the gate template was highlighted by bold numbers.

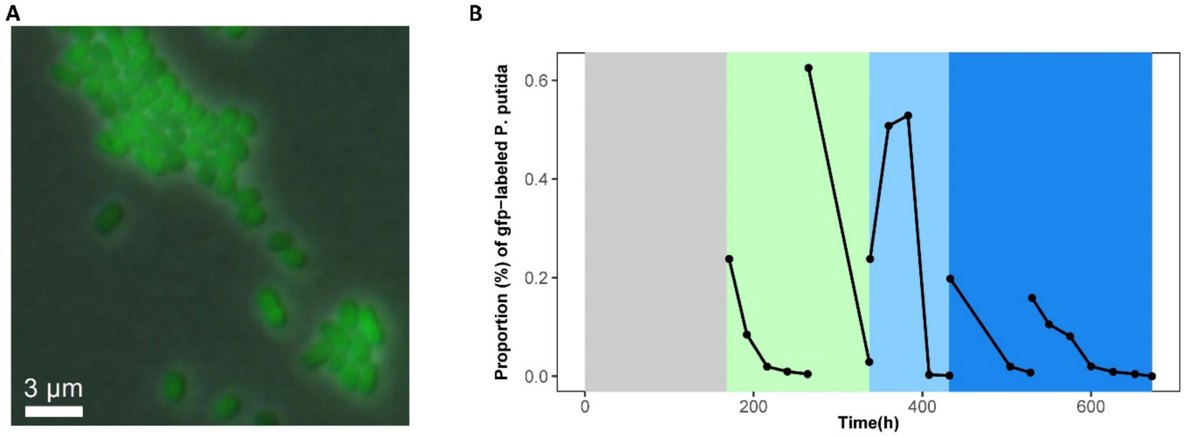

Figure S4.3: Green fluorescent *gfp*-labeled *P. putida* KT2440 (left) and loss of relative cell numbers of the strain after augmentation in the continuous bioreactor. The experiment was sub-grouped into four phases: grey: adaptation, green: *gfp*-labeled *P. putida* KT2440 augmentation, light blue: fosfomycin 10 mg L-1, blue: fosfomycin 268 mg L^{-1}.

Movie S4.1: Flow cytometric measurement of continuously grown cells in the bioreactor with regard to FSC, DAPI and GFP. GFP events are highlighted in red. The gate template is superimposed over all histograms.

Movie S4.2: Flow cytometric measurement of continuously grown cells in the bioreactor with regard to FSC, DAPI. The position of the cell gate and the gates for calibration beads are shown at the start of the movie. Time points for augmentation of *gfp*-labeled *P. putida* KT2440 and the various fosfomycin concentrations are highlighted.

S5: Diversity calculations

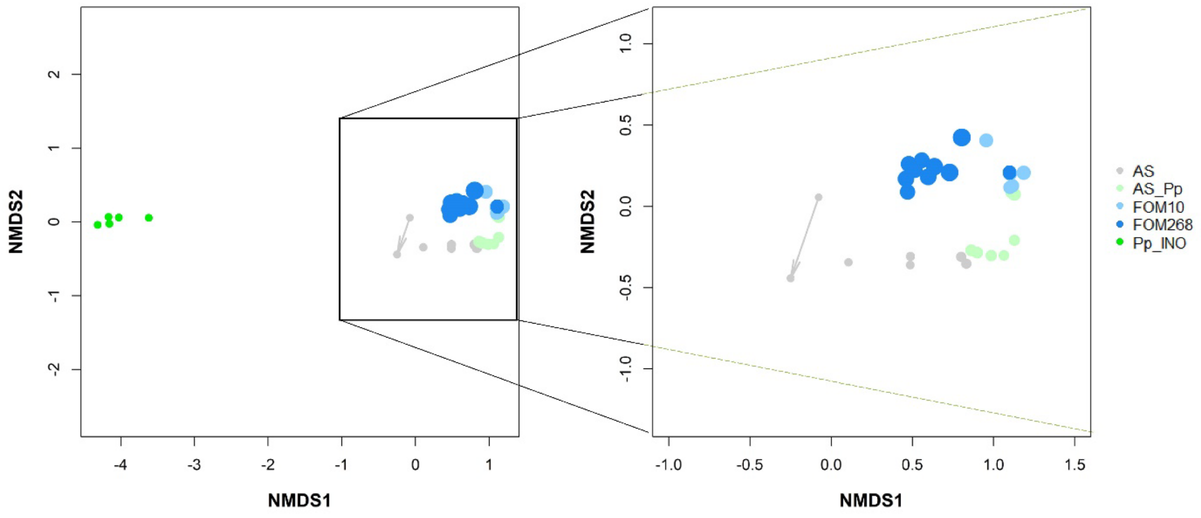

Figure S5.1: Dissimilarity analysis of the inoculated *gfp*-labeled *P. putida* KT2440 (green dots on the left side, Pp_INO) and the continuous cultivated WWC (colored dots on the right side). The experiment was sub-grouped into for four phases: grey (AS): adaptation, light green (AS_Pp): *gfp*-labeled *P. putida* KT2440 augmentation, light blue (FOM10): fosfomycin 10 mg L^{-1}, blue (FOM268): fosfomycin 268 mg L^{-1}.

	G09	G08	G01	G21	G29	G18	G04	G31	G30	G28	G15	G25	G22	G14	G27	G19	G20	G17	G24	G26	G13	G07	G12	G23	G16	G11	G05	G02	G10	G03	G06	α-diversity	β-diversity
1			●	●		●	●			●	●		●	●				●					●									10	
25	●		●							●		●		●	●		●	●	●													9	11
49			●									●	●	●	●		●	●	●			●										9	4
71													●	●			●	●		●	●	●	●	●								9	8
95												●	●	●	●		●	●	●	●	●	●	●	●								12	3
168						●							●	●						●	●	●	●	●	●							9	7
171						●							●	●						●	●		●	●	●							8	1
192						●							●							●	●			●	●	●						7	3
216						●							●								●			●	●	●					●	7	2
240													●							●	●	●	●	●	●							7	6
264												●	●	●			●			●	●		●	●	●							9	4
265												●	●	●			●			●	●	●	●	●								9	2
337													●	●						●	●			●	●		●		●		●	9	8
338													●	●						●	●			●	●		●		●		●	9	0
360													●	●							●			●	●		●		●		●	8	1
383													●								●			●	●	●	●	●	●		●	9	3
408						●	●														●			●	●	●	●	●	●		●	10	3
432													●	●							●			●	●	●	●	●	●		●	10	4
433													●	●							●			●	●	●	●	●	●		●	10	0
504						●					●	●	●	●	●						●						●		●	●	●	11	9
528												●	●	●							●								●	●	●	7	4
530												●	●	●	●						●						●		●	●	●	9	2
**550**												●	● (red)	● (red)	●						●						●		● (red)	●	●	9	0
575					●							●	●	●	●						●								●	●	●	9	2
600												●	●	●				●			●		●				●		●	●	●	10	5
626												●	●	●							●						●		●	●	●	8	2
652												●	●	●						●	●		●			●	●		●	●	●	11	3
672													●							●	●		●				●		●	●	●	8	3

Figure S5.2: Calculation of the α-diversity values and intra-community ß-diversity values (right column) of the WWC in the continuous bioreactor cultivation. The black points mark the subcommunities that were present (relative abundance >3.23%) in the WWC at the different time points during operation of the reactor (672 h, left column). The red points mark the subcommunities that were sorted. The experiment was sub-grouped into for four phases: grey: adaptation, green: *gfp*-labeled *P. putida* KT2440 augmentation, light blue: fosfomycin 10 mg L-1, blue: fosfomycin 268 mg L^{-1}.
